# Material extrusion 3D-printing technology: A new strategy for constructing water-soluble, high-dose, sustained-release drug formulations

**DOI:** 10.1016/j.mtbio.2024.101153

**Published:** 2024-07-14

**Authors:** Zhiting Liu, Jiaying Huang, Danqiao Fang, Bohua Feng, Jianxu Luo, Peixuan Lei, Xiaoling Chen, Qingchun Xie, Meiwan Chen, Peihong Chen

**Affiliations:** a State Key Laboratory of Quality Research in Chinese Medicine, Institute of Chinese Medical Sciences, University of Macau, Macau, China; b Department of Pharmaceutics, China Pharmaceutical University, Nanjing, 210009, China; c Center for New Drug Research and Development, Guangdong Pharmaceutical University, Guangzhou, 510006, China; d School of Traditional Chinese Medicine, Guangdong Pharmaceutical University, Guangzhou, 510006, China; e Guangdong Province Engineering & Technology Research Center for Medical 3D Printer and Personalized Medicine, Guangzhou, 510006, China; f YUEBEI People’s Hospital, Shaoguan, 512026, China

**Keywords:** Material extrusion, Direct ink writing, Metformin hydrochloride, Core-shell system, High drug loading, Sustained release

## Abstract

The advantage of low-temperature forming through direct ink writing (DIW) 3D printing is becoming a strategy for the construction of innovative drug delivery systems (DDSs). Optimization of the complex formulation, including factors such as the printing ink, presence of solvents, and potential low mechanical strength, are challenges during process development. This study presents an application of DIW to fabricate water-soluble, high-dose, and sustained-release DDSs. Utilizing poorly compressible metformin hydrochloride as a model drug, a core-shell delivery system was developed, featuring a core composed of 96 % drug powder and 4 % binder, with a shell structure serving as a drug-release barrier. This design aligns with the sustained-release profile of traditional processes, achieving a 25.8 % reduction in volume and enhanced mechanical strength. The strategy facilitates sustained release of high-dose water-soluble formulations for over 12 h, potentially improving patient compliance by reducing formulation size. Process optimization and multi-batch flexibility were also explored in this study. Our findings provide a valuable reference for the development of innovative DDSs and 3D-printed drugs.

## Graphical abstract

**Figure:**
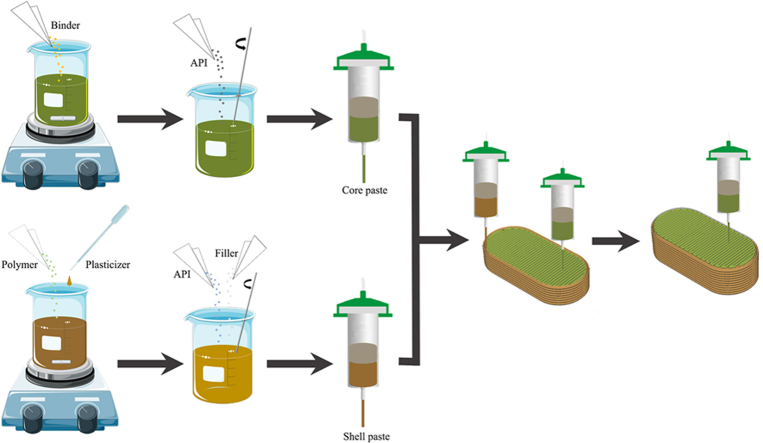

## 1 Introduction

3D printing is a transformative technology in fabricating drug delivery systems (DDSs), enabling the programmable release behavior of controlled microstructures. The development of microstructure in 3D printing has made it possible for oral DDSs to deliver the correct drug dose to the precise gastrointestinal location at the right time [1]. Since the Food and Drug Administration (FDA) approved the first 3D-printed drug, SPRITAM® (levetiracetam), in 2015, there has been an explosion of research into 3D-printed drug delivery systems (3DP-DDSs) [2,3], but no other drugs have yet received approval. Recently, the 3D-printed drug dosage forms of Triastek^@^ received Investigational New Drug approval from both the FDA and the National Medical Products Administration [4,5]. This regulatory endorsement underscores the promising role of 3D printing in the development and production of improved dosage forms, indicating its benefits for drug manufacturing. However, cost-effectiveness, laws and regulations, technical and regulatory constraints, and limited materials remain challenges in the industrialization of 3DP-DDSs [1,6,7]. We believe that combining compliant excipients with 3D printing technology to construct DDSs that cannot be produced using conventional processes represents the optimal utilization of its technological advantages and could be the best solution for industrialization opportunities. Therefore, investigating the ultimate limits of compliant excipients within 3D printing is crucial for unlocking the full manufacturing potential of 3DP-DDSs, thereby consequently accelerating the path toward industrialization.

Powder-based and material extrusion 3DP-DDSs are commonly used in the pharmaceutical industry. Material extrusion is mainly based on fused deposition modeling (FDM) and direct ink writing (DIW), of which FDM has a longer history and is more researched [8,9]. FDM-based technology has pioneered the feasibility of industrialization, the high temperature during the molding process, and the need for a large amount of polymer support pose challenges to filament preparation, thermal stability, crystal transformation, and drug loading [10]. Triatek^@^ has independently developed the MED^@^ technology, which is based on powder materials combined with FDM to eliminate the burden of filament preparation [11,12]. Conversely, DIW, a newly developed drug 3D-printing technology, employs room temperature or low temperature–molding methods to reduce the thermal stability burden of active pharmaceutical ingredients (APIs) [13]. However, as a nonthermal and non-compressive manufacturing process, DIW can also result in low mechanical strength, thereby necessitating the use of solvents requiring drying processes to form the final preparation [14]. Furthermore, solvent residues and solvent evaporation may damage the structural integrity due to cracking and shrinkage of preparations [15]. Moreover, solvent evaporation maintains the volume of the formulation, which can easily create a loose microchannel structure unfavorable for the controlled release of water-soluble drugs [16]. Despite the challenges associated with DIW, its mild molding conditions have brought new processes to the pharmaceutical industry, necessitating solutions to accelerate the development and industrialization of DIW drug manufacturing technology.

Sustained release of soluble drugs is a key issue in the pharmaceutical industry [17]. The development of insoluble polymeric excipients as drug carriers [18], complex structural designs such as core-shell [19], janus [20], and blank coating [21], and the combinations of core-shell and janus are effective strategies. However, these studies mostly focus on nano-DDS or drug delivery in multiunit systems and still require a significant amount of excipients. The design of a single-unit, high drug-loading, water-soluble, sustained-release drug delivery system has been a challenge. Metformin hydrochloride (MH), a first-line oral drug to treat type 2 diabetes mellitus, belongs to BCS Class III, meaning it is easily soluble in water, has a short half-life, and can be administered orally 4–5 times daily as high-dose tablets (500–1000 mg per tablet) [22]. The development of solid oral DDSs with sustained-release properties has attracted significant attention as they facilitate the maintenance of optimal plasma drug concentrations over an extended period, thereby decreasing the frequency of administration and enhancing adherence to the treatment regimen [23,24]. Although the sustained-release preparations have numerous advantages, formulating such products is challenging for high-dose and soluble APIs due to rapid and/or abrupt drug release [25]. High doses of MH can cause adverse effects such as vomiting, diarrhea, and gastrointestinal issues including acidosis [22]. Moreover, MH exhibits very poor flowability and tends to aggregate during storage, limiting the feasibility of tablet compression technology to produce high drug-loading formulations [26,27]. To achieve a good sustained-release curve for highly soluble drugs, a large amount of release-control polymer is required during tablet pressing. This results in the MH sustained-release tablets typically weighing approximately 1000 mg and having a large volume, which negatively impacts patient acceptance and compliance, especially in the elderly population who have trouble swallowing [28].

Keeping in mind the current scenario, we developed a single-unit, high drug-loading, sustained-release MH core-shell structure (MH-CSS) to reduce the volume and improve patient compliance with the medication. DIW and structural design were used to develop MH-CSS, where the core structure (MH-CS) contained almost only MH, thereby reducing the volume of high-dose formulations. In addition, the shell structure (MH-SS) was printed externally to block the potential burst release of tablets with high drug loads. To the best of our knowledge, this study is the first of its kind in which MH-CS with ultra-high drug-loading capacity has been prepared to achieve integrated molding of MH-CSS. This strategy provides an innovative solution for the delivery of high-dose, water-soluble drug formulations, thereby achieving sustained drug release while reducing the formulation volume. The study also demonstrates the process and potential of DIW for developing and optimizing DDSs, which will enrich the practice and theory of DIW in constructing innovative DDSs.

## 2 Materials and methods

### 2.1 Materials

MH was obtained from Jiuding Chemical Technology Co. Ltd. (Shanghai, China). MH standard was purchased from the National Institutes for Food and Drug Control (Batch No. 100338–201806). Polyvinylpyrrolidone (PVP) k90 was sourced from Star-Tech & JRS Specialty Products Co. Ltd. (Chongqing, China). Triethyl citrate (TEC) was obtained from Shanghai Macklin Biochemical Technology Co. Ltd. Eudragit® RS (PO) and Eudragit® RL (PO) were supplied by Shanghai Chineway Pharmaceutical Excipients Technology Co. Ltd. All additional chemicals and solvents used in this study were of analytical grade and were used without subsequent purification.

### 2.2 Methods

#### 2.2.1 Preparation of the MH core structure (MH-CS) paste and MH shell structure (MH-SS) paste

**Fig. 1:**
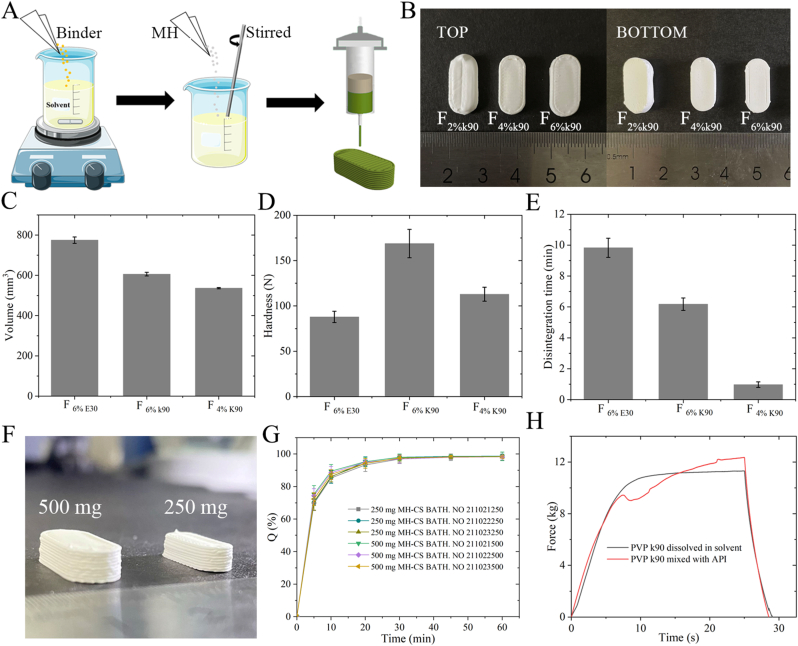
Preparation and printing process of paste ink (A), appearance (B), volume (C), hardness (D), and disintegration time (E) of MH-CS, and appearance (F) and dissolution study (G) of MH-CS with 250-mg and 500-mg specifications, (H) influence of the order of addition of PVP k90 simulated using a texture analyzer on the extrusion behavior of paste ink.

MH-CS paste: Configuration of the MH-CS paste is shown in Fig. 1A, and the formulations of MH-CS are listed in Table 1. To achieve high loading capacity and smooth extrusion, MH powder and excipients were pulverized and passed through a 125-μm mesh. The prescribed amount of the binder was first dissolved in a solvent and MH powder was added under high-speed stirring until a phase-homogeneous paste was formed. The prepared paste was loaded into a cartridge and subsequently secured with a seal.

**Table 1:** MH-CS formulations.

	Dose (mg)	Solvent	Adhesive	Binder (w/w)	MH (w/w)
F_6%E30_	500	90 % ethanol	HPMC E30	6.0 %	94.0
F_6%k90_	500	Ethanol	PVP k90	6.0 %	94.0
F_4%k90_	500	Ethanol	PVP k90	4.0 %	96.0
F_2%k90_	500	Ethanol	PVP k90	2.0 %	92.0

MH-SS paste: The prescribed amount of the plasticizer (Table 2) was added to an Erlenmeyer flask and mixed uniformly. The target dose of Eudragit® was added slowly while stirring at 200 rpm at 37 °C until it was well dispersed. The polymer was sealed for 4 h to allow complete swelling. The porogen and filler were added and mixed to produce a phase-homogeneous paste. The paste was then loaded into a print cartridge and sealed.

**Table 2:** MH-SS formulations.

	Solvent	Plasticizer	Plasticizer (w/w)	Eudragit® RS PO (w/w)	Eudragit® RL PO (w/w)	Talc (w/w)	MH (w/w)
F_Non MH_	95 % Ethanol	TEC	9.2 %	18.4 %	18.40 %	50.0 %	0
F_RS_				36.8 %	0	50.0 %	4.0 %
F_S1L1_				18.4 %	18.4 %	50.0 %	4.0 %
F_S1L2_				12.3 %	24.5 %	50.0 %	4.0 %
F_S1L4_				7.4 %	29.4 %	50.0 %	4.0 %
F_RL_				36.8 %	0	50.0 %	4.0 %
F_S1L4-TEC_				7.4 %	29.4 %	50.0 %	4.0 %
F_S1L4-PEG_		PEG 400		7.4 %	29.4 %	50.0 %	4.0 %
F_S1L4-GLY_		Glycerol		7.4 %	29.4 %	50.0 %	4.0 %

#### 2.2.2 Digital model and 3D printing

**Fig. 2:**
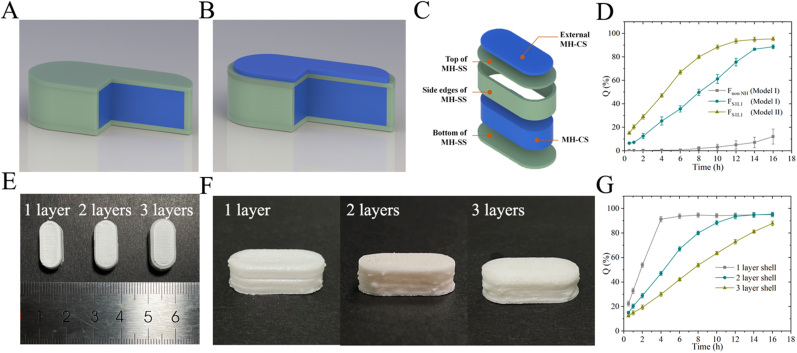
Design of MH-CSS (A), Model Ⅰ without an external MH-CS layer (B). Model Ⅱ with an external MH-CS layer (C). Explosion diagram of Model Ⅱ (D). Influence of pore formers and external layers on dissolution (E). Appearance of different MH-SS layers of MH-CSS and its side-enlarged view (F). Dissolution results (G).

The digital design (Fig. 2A and B) was crafted utilizing SolidWorks 2020 and subsequently saved in the. stl format for further processing. The file was subsequently imported into Cura software for slicing, resulting in a model that was exported with a. gcode file extension. The slicing parameters are shown in Supplementary Table 1. The pastes were loaded into the Auto Bio 2000 Bioprinter (Shenzhen Yuanyi Intelligence Pharma Tech Co. Ltd., China), which was then imported into the. gcode extension file. The tablets were dried in a well-ventilated oven initially at 45 °C for a duration of 2 h, followed by further drying at 80 °C for 6 h.

#### 2.2.3 Characterization of 3D-printed tablets

Shrinkage: Six tablets were randomly selected for measurement of their thickness and axes dimensions using precision digital calipers.

Hardness: The tablets were subjected to mechanical stress during the manufacturing process, transport, and drug distribution. According to the United States Pharmacopeia (USP) general chapter<1217>, six 3D-printed tablets were selected at random for hardness evaluation using a tablet hardness tester. Hardness values, expressed in Newtons (N), were documented and the mean values were computed.

Weight variation: Ten 3D-printed tablets were weighed individually on an electronic scale and the mean weight was determined. The content of MH was determined using high-performance liquid chromatography (HPLC) employing a Kromasil C18 column (4.6 mm × 250 mm, 5 μm) at a temperature of 30 °C. Chromatographic detection was conducted at a wavelength of 218 nm with an injection volume set to 10 μL. The mobile phase was composed of acetonitrile and phosphate buffer in a 900:100 (v/v) ratio, delivered at a flow rate of 1.0 mL/min (0.5 g each of sodium 1-heptane sulfonate and sodium chloride dissolved in water and diluted to 1000 mL and shaken well. The pH was adjusted to 3.85 using 0.06 mol/L phosphoric acid).

#### 2.2.4 Dissolution test

According to the USP-NF2021 dissolution method for MH extended-release tablets, dissolution testing was conducted using a paddle apparatus (CH-4147, SOTAX, Switzerland). Dissolution tests were conducted in hydrochloric acid solution adjusted to pH 1.2 and phosphate buffer solution at pH 6.8, with the samples agitated at a speed of 100 rpm and maintained at a temperature of 37.0 ± 0.5 °C. The method was as follows: at 0.5, 1, 2, 4, 6, 8, 10, 12, 14, and 16 h, 10 mL of the sample was withdrawn and the medium was replenished with an equal volume (10 mL) of fresh medium at the same temperature. After dilution of the sample, the MH content in samples was determined using ultraviolet spectrophotometry (UV-2700, Shimadzu) at a wavelength of 233 nm, and the cumulative drug release at each time point was computed.

#### 2.2.5 Drug-release kinetics

The release kinetics of MH from the formulation were evaluated through the application of zero-order, first-order, Higuchi, and Korsmeyer–Peppas models [29]. The model that best described the drug release was identified based on the highest correlation coefficient (R^2^) values.

#### 2.2.6 Swelling test

The polymer shell structure has been used to restrict the diffusion of water-soluble APIs. In addition to the shell thickness, microchannel formation within the shell is an important rate-limiting step for drug diffusion. The swelling behavior of MH-SS may have an impact on drug dissolution; consequently, a swelling assay was conducted to assess the impact of MH-SS on the drug dissolution profile.

**Fig. 3:**
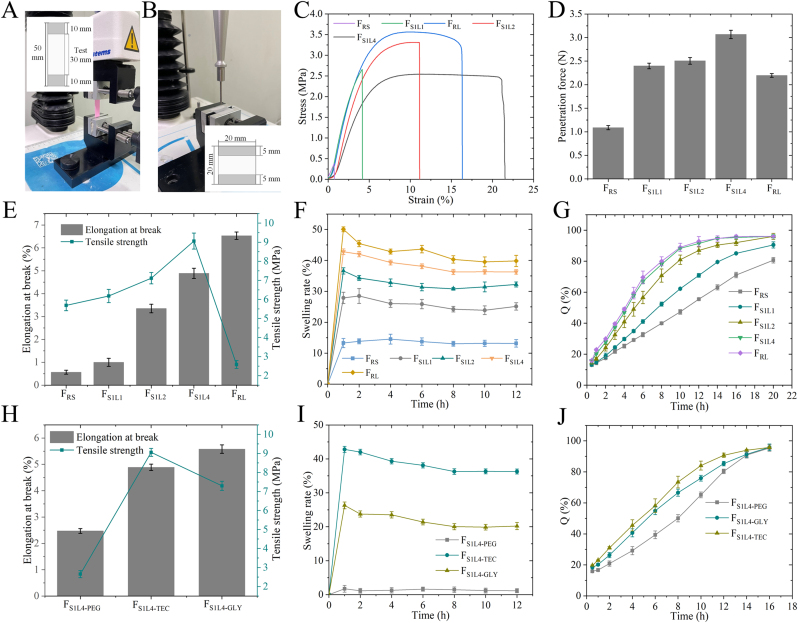
Optimization and evaluation of MH-SS. (A) Schematic of tensile test. (B) Schematic of puncture test. (C) Tensile test curve of different proportions of RL and RS. (D) Penetration force of different proportions of RL and RS. (E) Tensile strength and elongation at break of different proportions of RL and RS. (F) Swelling ratio of different proportions of RL and RS. (G) Dissolution results. (H) Effect of plasticizer type on tensile strength and elongation at break. (I) Effect of plasticizer type on swelling ratio. (J) Effect of plasticizer type on dissolution.

The MH-SS paste was printed into a 1 × 3–cm double-layer structure (Fig. 3A). After drying, the accurate weight (W_0_) was measured, and the sample was subjected to dissolution test conditions and removed after 1, 2, 4, 6, 8, 10, and 12 h. Residual solution on the surface was removed using filter paper, and the weight (W_t_) at each designated time point was documented. The swelling ratio (SR) at each time interval was determined in accordance with Equation (1).

(1) $$SR=\frac{W_{t}-W_{0}}{W_{0}}\times 100\%$$

where W_0_ is the initial mass (mg) and W_t_ is the mass after swelling (mg).

#### 2.2.7 True density

The gas volumetric method for testing the volume of porous materials is closer to the actual volume of the sample [30]. The MH-CSS and reference preparations were placed separately in the chamber of a true density instrument (AccuPyc II 1340, Micromeritics, American), and helium was used as the measuring gas. The pressure within the chamber was incrementally increased to a predetermined level of 19.5 psi, permitting helium to permeate into the chamber. The equilibrium pressures achieved during both processes were automatically documented. From the pressure before and after gas diffusion, the volume and true density of the sample were calculated using Boyle–Mariotte law.

#### 2.2.8 Differential scanning calorimetry (DSC)

DSC was conducted for the API, excipients, physical mixtures (PMs), and final preparations using a DSC 4000 instrument (PerkinElmer, USA). Samples were sealed in aluminum pans and heated from 30 to 300 °C at a rate of 10 °C/min under a nitrogen atmosphere at a flow rate of 20 mL/min. Prior to testing, the sample temperature was maintained at 30 °C for 10 min and the sample mass was accurately weighed to 5 mg.

#### 2.2.9 Fourier-transform infrared (FT-IR) spectroscopy

FT-IR spectroscopy was used to analyze the API, excipients, PMs, and formulated preparations. Sample pellets were prepared by combining 2 mg of the sample with 100 mg of dried KBr, ground, mixed uniformly, and pressed into a smooth, transparent pellet. A TENSOR 27 spectrophotometer (Bruker, Germany) was used to obtain spectra in the wavenumber range of 400–4000 cm^−1^, and each sample was scanned three times for consistency.

#### 2.2.10 X-ray powder diffraction (XRD)

XRD patterns for the API, excipients, PMs, and MH-SS preparations were generated using an ULTIMA IV diffractometer (Rigaku, Japan) equipped with a copper target. The tube voltage was set at 40 kV and the current at 50 mA. The diffraction angles (2θ) were scanned from 5° to 50° at a rate of 6°/min.

#### 2.2.11 Texture analysis

Tensile test: The tensile test of the texture analyzer was used to evaluate the tensile properties and stability of the MH-SS and to determine whether the swelling pressure or high internal osmotic pressure of the polymer during the dissolution process of the MH-CSS would cause the MH-SS to break. The sample was prepared as a strip as shown in Fig. 3A, subjected to dissolution test conditions, and removed after 12 h. Tensile testing was performed using a TA-XT plus texture analyzer equipped with an A/TG probe (Pekin Elmer, America). The impregnated MH-SS was tensioned at equal lengths from top to bottom, with a 30-mm reserve in the middle. The elongation at break (F_b_) of the film was measured at a tensile speed of 0.5 mm/s, and tensile strength (σ) was calculated using Equation Ⅱ:

(2) $$\sigma =F_{b}/S_{o}$$

where *F*_*b*_ is the maximum force the strip film could withstand when stretched (expressed in N) and *S*_*o*_ is the cross-sectional area of the strip (expressed in mm^2^). The unit of tensile strength is expressed in MPa.

Puncture test: The hardness of the MH-SS was assessed using the puncture test. The MH-SS was printed in a strip form with two layers as shown in Fig. 3B, subjected to dissolution test conditions, and removed after 12 h. MH-SS was placed on the A/TG base, leaving 10 mm in the center, and a P/2 probe was used for testing. The test speed was 1.0 mm/s, and the test was stopped when the puncture displacement was 10 mm. The puncture force was recorded. The number of samples was three.

Hardness: As the hardness of MH-CSS exceeded the upper limit of the hardness tester (>200 N), a texture analyzer was used to determine the hardness of 3D-printed MH-CSS. Six tablets of the reference formulation (Glucophage® XR, Merck KgaA) and 3D-printed MH-CSS were placed vertically directly under the P/6 probe of the texture analyzer, and the maximum load forces were tested in the radial direction at a test speed of 1.0 mm/s.

#### 2.2.12 Field emission scanning electron microscopy (FE-SEM)

FE-SEM uses high-energy electron beams to image samples by faster scanning, providing a microscopic image of the surface morphology of solids. The preparations were fixed on an aluminum disc with conductive carbon, and its surface was evenly sprayed with gold powder to increase conductivity. A Merlin FE-SEM equipment (Zeiss, Germany) was used to observe and acquire images.

## 3 Results and discussion

### 3.1 Design principles of formulations

We constructed an ultra-high drug-loading, sustained-release, and smaller-volume preparation by adopting the concept of DIW combined with modular design, thereby providing a new strategy for constructing a delivery system for the delivery of high, single-dose administration and sustained release of APIs. Our formulation also demonstrates the potential of DIW in building innovative DDSs. Accordingly, we designed a DDS for MH-CSS (Fig. 2B and C). MH-CS is composed of a small amount of binder and has ultra-high API loading. MH-SS is designed as a multilayer shell structure to withstand the high osmotic pressure of MH-CS and gastrointestinal peristalsis while avoiding burst release of the drug. This shell structure can also cause a delay in drug release. Based on the modular design concept, we moved one layer of the MH-CS to the top of MH-SS to compensate for the potential release delay in the early stages. In summary, we explored additive manufacturing technology as a strategy to design a system with ultra-high drug loading for the sustained release of water-soluble APIs and have demonstrated how formulation optimization and modular design concepts can be used to achieve API release based on predetermined requirements.

### 3.2 Optimization and characteristics of MH-CS

MH has poor fluidity and is prone to aggregation, making it challenging to prepare high drug-loading formulations using traditional processes [26]. We believe that DIW requires the preparation of excipients and API particles into a paste, with high polymer chains and high viscosity systems to avoid the agglomeration of MH particles and provide lubrication, which is beneficial for the design of ultra-high drug-loading formulations. To maximize the drug-loading efficiency of the MH compound in the controlled system (CS), the formulation exclusively incorporated binders as excipients, with the drug-loading capacity standardized to 500 mg. The properties of MH-CS have a crucial impact on its formation and final quality. The first step involved its optimization and characterization, and Fig. 1A shows the process used for MH-CS preparation. The digital model, cut model, printing process, and cutting parameters are shown in Supporting Fig. 1 and Supporting Table 1.

We performed a simple screening of two classic binders, HPMC E30 and PVP k90, and found that both had complete structures after molding when added at 6 % (w/w) (Supplementary Fig. 2). The volume of F_6%k90_ decreased by 21.8 % compared with F_6%E30_ (Fig. 1C), but its hardness increased by 92.0 % (Fig. 1D), indicating that the space occupied by MH was higher in the F_6%k90_ preparation. The observed phenomenon could be attributed to the distinct binding interactions characterizing HPMC E30 and PVP K90. Water is required for HPMC to exert its binding effect, wherein the molecular chains swell and act as a solid bridge between the API particles. After drying, HPMC shrinks, creating more voids in the tablet, resulting in a larger volume and lower hardness. PVP k90, on the other hand, dissolves better in solvents, allowing more contact between API particles and providing a more robust bridging effect. Fig. 1D shows that the disintegration rate of F_6%E30_ to be 59 % slower than that of F_6%k90_ (Fig. 1D), further supporting the above view. After contact with water, F_HPMC E30_ swelled and dissolved, preventing water from penetrating the micropores of the tablet core, resulting in a lower disintegration rate and density than F_PVP k90_. This phenomenon also occurs during the coating of conventional tablets. To further increase the drug loading of MH-CS, we attempted to further reduce the binder quantity. F_2%K90_ with 2 % (w/w) binder was printed, but there were structural defects (Fig. 1B). Although the hardness of F_4%K90_ was 33.1 % lower than F_6%K90_, the value still reached 112.9 ± 7.7 N (Fig. 1D), thereby meeting storage and transportation requirements. Meanwhile, reducing the binder by 2 % (w/w) resulted in a volume reduction benefit of 11.5 % (Fig. 1C). This approach met our optimization expectations for MH-CS, wherein a smaller volume could be used without compromising the drug content. However, this posed a challenge because the disintegration time of F_4%K90_ was only 0.97 ± 0.18 min, which is 5.4 times faster than that of F_6%K90_ (Fig. 1E). This finding suggested rapid hydration and high osmotic pressure in the core of MH-CSS, warranting attention to the structural design of subsequent MH-SS.

The 250-mg and 500-mg specifications of MH-CS were prepared for characterization and evaluation, both of which exhibited a relatively perfect appearance (Fig. 1F) with the actual size being almost consistent with the digital model (Supporting Table 2). The actual weights of the 250-mg and 500-mg MH-CS tablets were 269.80 ± 3.20 mg and 521.10 ± 4.20 mg, respectively (Supporting Table 3). To verify the reliability of the process and formulation, three batches of MH-CS were prepared and extensively evaluated. The drug contents in the 250-mg and 500-mg specifications of MH-CS tablets (Supporting Table 4 and Supporting Table 5) were 255.11 ± 0.85 (relative standard deviation [RSD] = 0.33 %) and 500.37 ± 0.63 (RSD = 0.13 %), respectively, and drug loading was as high as 95.96 ± 0.60 (RSD = 0.63 %) and 95.56 ± 0.28 (RSD = 0.29 %), respectively (Supporting Table 4 and Supporting Table 5). The inter-batch differences in the three batches were significantly lower than the requirements stated in the Chinese Pharmacopeia. Moreover, MH from MH-CS dissolved completely within 30 min and showed a similar behavior (Fig. 1G). The differences between batches and the excellent quality of MH-CS cannot be separated from the key process attributes. The sequence in which PVP k90 was added is a key process parameter. We found that PVP k90 as an adhesive should first be dissolved in the solvent and then mixed with MH to form a paste ink, rather than premixing with MH. Incorrect mixing methods can lead to large fluctuations in extrusion pressure and cause uneven extrusion volume, thereby affecting product quality. We used texture analysis to simulate the extrusion process and found that the correct method of material addition led to a smooth extrusion process, whereas incorrect methods resulted in the inability of the paste to be extruded at a constant pressure and flow rate (Fig. 1H). The reason for this phenomenon was that PVP k90 was not mixed uniformly and was not sufficiently in contact with MH particles, thereby resulting in the formation of unstable and agglomerated particles in the paste-like ink. This unstable paste exhibited unstable flow and changes in the ultimate viscous resistance within the die, resulting in increases or fluctuations in the extrusion pressure. This finding is similar to our previous research conclusion that in extrusion-based 3D printing [31], the order of material addition is a key factor influencing the applicability and formability of extrusion-based 3D printing.

### 3.3 Design of MH-CSS

MH-CS with a 500-mg drug load exhibited a drug-loading capacity of up to 95.56 % and demonstrated a rapid disintegration rate with a mean time of 0.97 ± 0.18 min. We ensured the formation and mechanical strength of the formulation using minimal excipients, but this approach can also cause the immediate release of water-soluble MH. As described in Section 3.1, we adopted a core-shell structure format for the design concept, which gave the formulation a longer drug-diffusion path and a more robust structure (Fig. 2B and C). The initial design concept was to achieve sustained drug release through the self-induced microporous effect of the polymers. However, in contrast, the 16-h cumulative dissolution of F_non NM_ (Model Ⅰ) was <20 % (Fig. 2D), which may be related to the thicker diffusion path, fewer pores, and low permeability of MH. Therefore, we added MH to MH-SS to promote the formation of drug-release channels and increase the drug loading of the final preparation. Although the dissolution rate of F_S1L1_ (Model Ⅰ) significantly increased at 16 h, there was a serious delay in release, which may have been related to the decrease in MH-induced pore efficiency caused by the thicker MH-SS. We adopted a modular design concept and moved one layer of MH-CS to the outside of the tablet (Fig. 2B and C). The MH-release delay phenomenon disappeared and the drug release was more complete (Fig. 2C). However, appropriate strategies still need to be adopted to optimize drug release. According to the nozzle characteristics (Supplementary Table 1), the width of each layer of MH-SS was about 0.26 mm, which was significantly thicker than the micrometer thickness of conventional coatings. We adjusted the number of layers of MH-SS to 1, 2, and 3, and printed them into shape (Model Ⅱ). The number of layers in MH-SS did not affect the moldability of MH-CSS (Fig. 2E), but in the locally magnified image, structural defects can be seen on the side of MH-CSS in single-layer MH-CSS (Fig. 2F). This phenomenon may be due to insufficient interlayer adhesion during the layer-by-layer deposition process of 3D printing, or the low strength of the single-layer MH-SS structure, which was blasted by volatile solvents during the drying process. The monolayer structure affects MH-CS integrity and results in complete drug release within 4 h (Fig. 2G). The three-layer MH-CS showed slow and incomplete drug release. Therefore, a two-layer shell structure was adopted to ensure the structural integrity of the formulation and uniform drug release. All subsequent studies were conducted using Model Ⅱ (Fig. 2B and C) of the two-layer MH-CSS.

### 3.4 Optimization and evaluation of MH-SS

The polymer in the shell structure of MH-CSS was mainly composed of Eudragit® RL and Eudragit® RS, both of which can be self-porous, with pore sizes ranging from 1 to 5 μm and 0.1–0.6 μm [32,33], respectively. Optimizing the ratio of these two components is beneficial in obtaining better drug-release curves and improving the mechanical strength of MH-CSS. Formulations (Table 2) with different ratios of Eudragit® RL PO (RL) and Eudragit® RS PO (RS) showed excellent molding effects (Supporting Fig. 3). Double-layer MH-SS with different RL and RS ratios were printed based on the graphical parameters (Fig. 3A and B), and tensile and puncture tests were performed using a texture analyzer. The tensile strength was determined using Equation Ⅱ. The puncture resistance of MH-SS with different ratios of RL and RS was higher than that of RL or RS used alone (Fig. 3C, D, 3E), which revealed that DIW achieved a consistent effect with traditional processes and that the mechanical strength of films can be improved by using different types of resins mixed together. The higher the tensile strength, the greater the resistance of MH-SS to stretching, which favors resisting the high osmotic pressure of the tablet core and maintaining the structural integrity of the formulation during release. F_S1L4_ had the highest tensile strength (Fig. 3E) of 9.06 Mpa and puncture force (Fig. 3D) of 3.07 N, indicating that it was more conducive to resisting repeated stress shocks caused by gastrointestinal peristalsis. We also investigated the swelling behavior of MH-SS with different RL and RL ratios during the dissolution process. The RL ratio as well as the swelling rate of MH-SS increased. The increase in swelling rate was due to the higher content of quaternary ammonium substituents in RL, which resulted in stronger hydrophilicity and higher swelling capacity [34]. The high swelling rate also provided more microporous structures for MH-SS, thereby accelerating drug release. The dissolution test results (Fig. 3G) further corroborated these findings and showed a dissolution rate trend of F_RS_ < F_S1L1_ < F_S1L2_ < F_S1L4_≈F_RL_. The dissolution curves of the two surfaces were similar when the f2 value was >50. The closer the value was to 100, the higher the similarity [35]. The f2 values of F_S1L4_ and F_RL_ were 84, indicating similar dissolution behavior for the two, and also revealing that increasing the RL had little effect on the drug-release behavior. Therefore, after optimizing the formulation of RL and RS in different proportions, F_S1L4_ exhibited the most suitable drug-release behavior and the best mechanical strength.

Plasticizers lubricate the areas between polymer chains to improve the flexibility of the sustained-release film [36]. We analyzed the effects of different types of plasticizers (Table 2) on the performance of MH-SS, and all of them showed excellent formability (Supporting Fig. 4). Glycerol as a plasticizer could significantly reduce the tensile strength and swelling of MH-SS (Fig. 3I). At the same time, the dissolution rate of MH reduced significantly, with a 12-h dissolution rate of only 85.3 %, whereas the dissolution rate of F_S1L4-TEC_ was 90.7 %. This may be due to the stronger hydrogen bonding of glycerol in RL and RS, which limited the affinity and swelling of the polymer with water, thereby reducing the aperture of the channel and leading to slower drug release. This stronger hydrogen bond also significantly increased printing pressure (Supporting Table 6). The lower swelling degree and release rate of F_S1L4-PEG_ also supported this view. Overall, the use of TEC as a plasticizer led to the best mechanical strength (Fig. 3H), as well as making MH release gentler (Fig. 3J). After screening the amount of plasticizer, a ratio of TEC:RL and RS of 1:4 yielded the best product (Supporting Fig. 5). It also increased the elongation at break and puncture force of MH-SS by 171.6 % and 97.4 %, respectively, compared with the formulation without the plasticizer (Supporting Fig. 6). When the amount of TEC was further increased, the tensile strength was reduced by 30.5 %. Overall, we investigated the effects of formulations and processes on MH-SS formability and MH release to provide a reference for DIW design and formulation optimization.

**Fig. 4:**
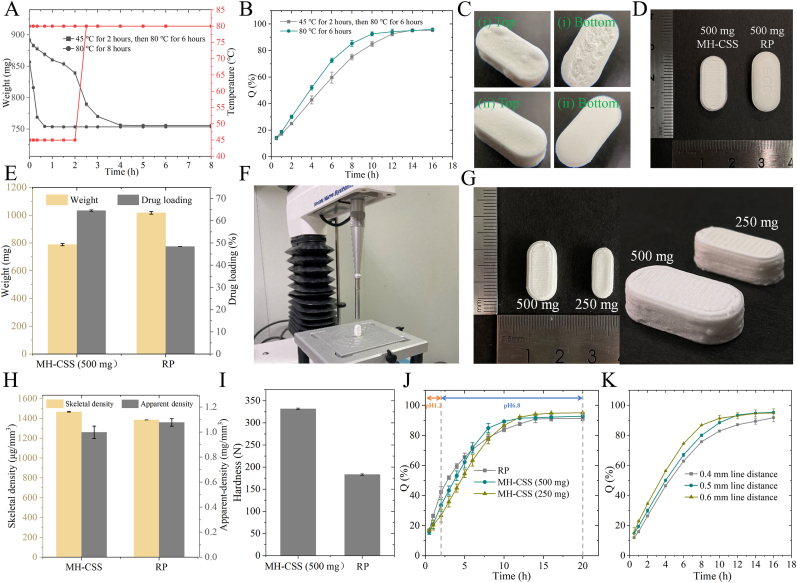
Evaluation of MH-CSS and comparison with reference preparations. (A) Drying method and its effect on tablet weight. (B) Effect of drying process on dissolution. (C) Influence of drying process on the appearance of MH-CSS. (D) Appearance of MH-CSS and RP. (E) Weight and drug content of RP and MH-CSS. (F) Schematic diagram and (I) Results of hardness testing of tablets using a texture analyzer. (H) Apparent density and skeletal structure CSS. (G) Appearance of 250-mg and 500-mg MH-CSS. (I) Hardness of MH-CSS and RP. (J) Dissolution of MH-CSS and RP. (K) Dissolution results of MH-CSS with different internal fill rates.

**Fig. 5:**
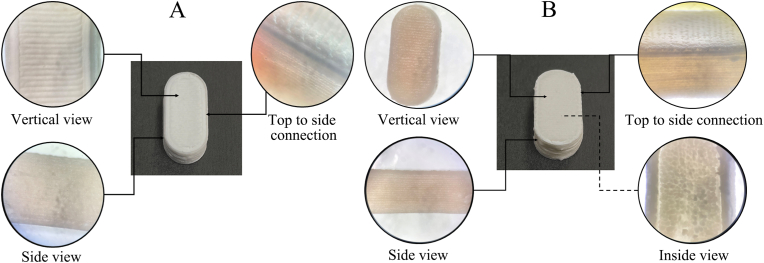
Comparison of appearances before dissolution (A) and after dissolution (B).

**Fig. 6:**
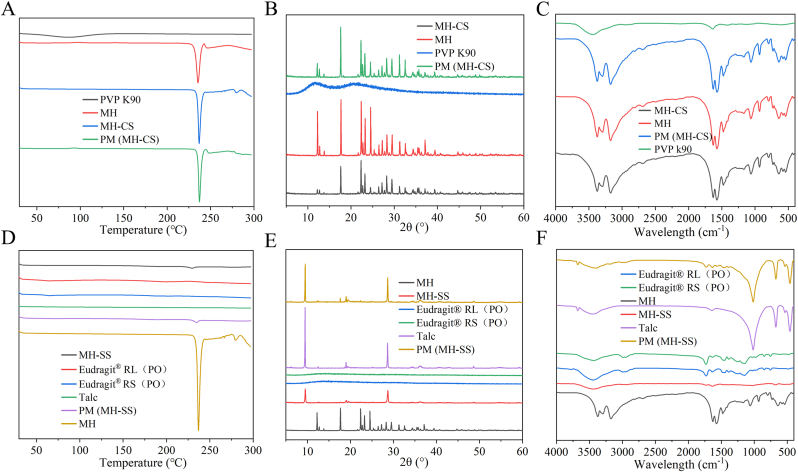
DSC (A), XRD (B), FT-IR (C) spectra of MH-CS, and DSC (D), XRD (E), FTIR (F) spectra of MH-SS.

### 3.5 Evaluation of the drying process

The addition of solvents and drying procedures are essential in the DIW molding process. Therefore, we investigated the effect of drying procedures on the moldability and quality of the dissolved MH-CSS. The thick shell structure of MH-CSS allowed for sustained drug release but prolonged the solvent-volatilization pathway. Inappropriate processes may result in bulging, cracking, or prolonged drying time. At a drying temperature of 80 °C, MH-CSS reached drying equilibrium within 1 h (Fig. 4A). However, bulges appeared on the surface of MH-CSS, and delamination occurred at the bottom due to excessive adhesion between the material and the platform (Fig. 4C i). This is because, at higher temperatures, the rate of solvent evaporation in MH-CS was greater than the rate of gas evaporation in MH-SS. To facilitate the rapid evacuation of excess gas, MH-SS underwent deformation to increase the gas-diffusion area, thereby forming bulges. The deformation and bulging phenomena also led to some shell structures becoming thinner or exhibiting an increased number of micropores, consequently accelerating the drug-release rate (Fig. 4B). Although the process was carried out at a constant temperature of 45 °C for 2 h followed by heating up to 80 °C, the appearance of MH-CSS was more satisfactory (Fig. 4C ii) when a drying time of 4 h was used (Fig. 4A),. Therefore, in the DIW process, where solvents cannot be avoided, it is necessary to not only pay more attention to the design and process optimization of the formulation but also to the post-treatment drying procedure.

### 3.6 Evaluation of MH-CSS and comparison with reference preparations

**Fig. 7:**
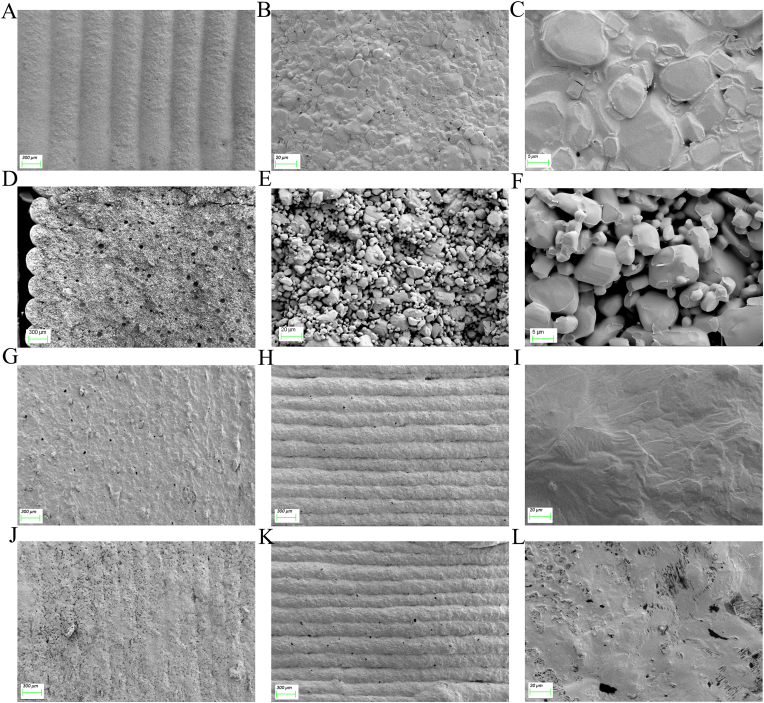
Surface (A), partial (B), and high-magnification (C) views of MH-CS. Surface (D), partial, (E) and high-magnification (F) views of MH-CS cross-section; surface (G), side (H), and side-magnification views (I) before MH-CSS dissolution; surface (J), side (K), and side-magnification views (L) after the dissolution of MH-CSS.

To compare MH-CSS prepared using DIW and traditional process formulations, we selected Glucophage^@^ SR as a reference formulation (RP) for consistency evaluation and comparative testing. MH-CSS was prepared as shown in Supporting Table 7. Visually, the volume of MH-CSS appeared smaller than that of the reference formulation (Fig. 4D). The surface of MH-CSS exhibited obvious 3D-printed streaks, whereas that of RP was smooth. The weight of MH-CSS was 787.6 ± 7.9 mg (Supporting Table 8) and that of RP was 1016.8 ± 9.4 mg, which corresponded to a weight reduction of 22.5 %. Three bath drug content of MH-CSS was found to be 64.6 % using HPLC (Supporting Table 11), which was 33.4 % higher than that of the RP at 48.4 %. The volume of MH-CSS was determined to be 699.8 ± 33.2 mm^3^, whereas that of RP was 943.3 ± 32.1 mm^3^. The use of the DIW technique resulted in a volume reduction of 25.8 %. Using Archimedes’ formula, the apparent densities of RP and MH-CSS were calculated to be 1.1 mg/mm^3^ and 1.0 mg/mm^3^, respectively. The densities of the two were close (Fig. 4H). MH-CSS had a smaller volume and weight but was produced by an uncompressed DIW process and underwent solvent evaporation. Theoretically, its internal structure is more porous and the product has lower mechanical strength. However, the hardness of the specimen surpassed the maximum measurable threshold of the hardness testing apparatus. Therefore, a texture analyzer was used for hardness analysis (Fig. 4F). The hardness of MH-CSS was 331.89 ± 1.68 N, which was 1.8 times that of the reference formulation (Figs. 4I) and 2.9 times that of CS (Fig. 1D). This high hardness was due to the direct action of the free adhesive on MH particles and the formation of a more stable solid bridge between particles, which can also be seen from SEM (Fig. 7C and F). Using the helium true-density measurement method and the ideal gas state equation, the volume of gas released by the material under specific temperature and pressure conditions can be calculated to obtain the true density of the material [37,38]. The skeletal density of MH-CSS was 1466.70 ± 2.40 μg/mm^3^, whereas that of RP was 1384.00 ± 1.00 μg/mm^3^ (Fig. 4H). Although the skeletal density of MH-CSS only increased by 6.5 %, its apparent density was lower. This finding indicated that MH-CSS had more voids inside than RP, but the density of the solid part was higher than that of RP. The high-density skeletal structure provided it with high hardness, which explained why its hardness was higher than that of RP. In addition to the solid bridging effect of the adhesive, the free MH resulting from solvent evaporation underwent interparticle recrystallization, further enhancing the microscopic strength of the solid skeleton structure. This also suggests that although DDSs prepared using DIW may have a lower apparent density, appropriate formulations and processes can be used to optimize the density or strength of their solid skeleton to compensate for the low mechanical strength resulting from the low apparent density.

Results from the release model fitting (Supplementary Table 12) indicated that 250-mg MH-CSS fitted the Korsmeyer–Peppas mathematical model with an R^2^ of 0.9939, whereas 500-mg MH-CSS was more consistent with zero-order release with an R^2^ of 0.9905. The fitted n values of the Korsmeyer–Peppas mathematical models were 0.6188 and 0.5802, respectively. A value of 0.45 < n < 0.89 indicated that drug release was the result of the combined action of Fick's diffusion and matrix corrosion. However, during the actual drug dissolution of MH-CSS, only rapid dissolution of the external MH-CS layer was observed, whereas the remaining structures remained consistent before and after drug release (Fig. 5A and B). This strategy of placing an external layer of MH-CS to complement the delayed release may interfere with the evaluation and exploration of the drug-release mechanisms. Therefore, the dissolution of Model I (Fig. 2A) without the external MH-CS layer was evaluated, and the drug release was found to be more consistent with zero-order release, with an R^2^ of 0.9809. The fitting result of the RP equation was n = 0.3985, which was less than 0.45. Taken together, these findings suggested that the drug release of MH-CSS (Model I) without an outer layer was mainly diffusion-oriented and that MH-SS provided a blocking effect on drug diffusion. When using Model II (Fig. 2B) to prepare MH-CSS, the outer layer complemented the previous release delay, but also interfered with the evaluation of the drug-release mechanism. This is a factor that should be considered when using a modular design approach to prepare complex structured formulations. In general, the drug-release mechanism in MH-CSS was achieved *via* the rapid disintegration of the outer layer to compensate for the delayed release in the early stages and by Fick's diffusion of drugs blocked by the core-shell structure. The drug-release model for RP was complexed with the Higuchi model and an R^2^ of 0.9923 was obtained. The drug-release mechanism can be attributed to the blocking effect of the gel layer on MH diffusion after swelling. Although the gel layer of RP swells to achieve sustained drug release, individual differences in gastrointestinal motility and eating habits may challenge the fate of the gel layer of RP *in vivo*. This can lead to significant differences in individual pharmacokinetics. The design concept of the noncorrosive core-shell structure of MH-CSS is more conducive to maintaining the integrity of the formulation *in vivo* and reducing individual pharmacokinetic differences. Based on computer-aided drug design, the fill rate of MH-CS has been adjusted to achieve personalized release behavior under the same dosage specifications. This does not require changing the formulation and processing of tablets but only changing the model parameters to meet the personalized needs of patients with special medication requirements (Fig. 4K). The concept of modularity and computer-aided design applied to DIW technology has enabled the manufacturing of high-dose sustained-release drug formulations and reduced volume, demonstrating the potential in the cross-application of new technologies for the development of DDSs.

### 3.7 DSC, FT-IR, and XRD

As an innovative strategy for the construction of DDSs, DIW requires solvents and drying processes that may induce potential interactions between raw materials and excipients, such as crystal transformation. Therefore, DSC, FTIR, and XRD were performed on MH-SS and MH-CS. DSC (Fig. 6A) indicated a sharp endothermic peak for MH at a temperature of 236.9 °C, which was also observed in PM and MH-CS without significant migration. Similarly, the MH peak in MH-SS underwent a slight shift compared with that of API, which may be due to hydrogen bond interactions. There is currently no evidence of crystal transformation of the API based on DSC. The main diffraction peaks (Fig. 6B and E) of MH in XRD were 12.2°, 17.7°, 22.3°, 24.5°, and 37.1°, and no significant changes were observed in MH-SS and MH-CS, indicating that there was no evidence of crystal phase transformation in the DIW process. The peak at 9.4° in the XRD spectrum of MH-SS represents the diffraction peak of talc (Fig. 6E). The absorption bands at 3375 cm^−1^ and 3298 cm^−1^ in the IR spectra are the asymmetric and symmetric stretching vibrations of –NH_2_; the absorption peak at 3170 cm^−1^ represents the stretching vibration absorption peak of –NH; and the absorption peak at 1627 cm^−1^ can be attributed to the stretching vibration of –C=N. Furthermore, the double absorption peak near 1476 cm^−1^ corresponds to the symmetric bending vibration absorption peak of the methyl –C-H (Fig. 6C and F). These peaks did not show significant changes in MH-CS, nor did new peaks appear or disappear. The characteristic peak of MH in MH-SS showed a slight redshift (Fig. 6F), which, together with the DSC results (Fig. 6D), indicated the possibility of hydrogen bonding between MH and the excipients, but no chemical reaction was found. Overall, no evidence of raw material incompatibility was found in MH-CSS, indicating the suitability of DIW to prepare MH-CSS. The introduction of solvents and the use of drying processes are not drawbacks in the production of DDSs by DIW. Although it prolonged the processing time, it did not cause incompatibility among raw materials. Moreover, it provided satisfactory mechanical strength and drug-release behavior of the delivery systems. This sustained-release behavior can be easily adjusted using a customized modular design approach.

### 3.8 Microstructure analysis of MH-CSS

The semisolid form of the preparation changes to the solid state after the solvent volatilizes. Solvent volatilization forms irregular cavities or defects inside, potentially affecting the mechanical strength of the DDS. In the MH-CS surface (Fig. 7B and C) and cross-section (Fig. 7E and F), MH was uniformly distributed in the crystal form and PVP k90 formed a solid bridge tightly connecting the MH particles. Although the stacking density of the internal MH particles was lower, the solid bridges between the particles were tightly connected, forming a stable skeleton structure inside. This accounted for the high drug loading of MH-CS at 112.9 N with 95.56 % efficiency. The numerous pores in MH-CS and the hydrophilicity between MH and PVP k90 easily led to the capillary effect (Fig. 7C and F), which is an important factor contributing to the rapid disintegration of MH-CS. MH-CSS adopts a core-shell DDS design philosophy; thus, the effect of dissolution on the structural fate of MH-CSS is a key consideration factor. In the macroscopic image, the formulation retains a distinct 3D-printed stripe structure before dissolution (Fig. 5A), which was also confirmed by SEM (Fig. 7A and H). The formulation displays tight connections at the sides and corners (Fig. 5A), and the microstructure shows the dense surface structure of MH-SS (Fig. 7G and I) with tight interlayer connections (Fig. 7H). Small microporous structures are visible in the interlayer and on the surface (Fig. 7G and H), which are not formed by the pore-forming agent but formed by the solvent evaporation channel during the drying process of MH-CSS to accelerate the solvent escape from MH-CS. The side view shows the interlayer bonds of MH-SS to be relatively thin and prone to defects (Fig. 7H). If only one layer of the shell structure is printed outside the MH-SS, this defect can lead to the rapid contact of the dissolution medium with the core, leading to accelerated drug release (Fig. 2G). This problem can be avoided by printing a 2-layer stacked shell structure (Fig. 2G). The microscopic images before (Fig. 7I) and after (Fig. 7L) dissolution of MH-SS show that the addition of MH as a pore-forming agent led to the formation of numerous micropores. This regulated MH release to achieve complete drug dissolution within 12 h. Microstructure analysis revealed the interaction between the API and excipients as well as the fate of MH-CSS before and after dissolution. These findings are beneficial in exploring the formation and release mechanisms of DIW-based preparations, providing in-depth analysis and reference for the development of 3D printing for high drug-loaded preparations.

## 4 Conclusion

This study innovatively applies DIW 3D printing to develop a core-shell DDS for MH, a water-soluble, high-dose drug. High drug-loading capacity with sustained release over 12 h was obtained, potentially enhancing patient compliance through reduced dosage form size. The study introduces a modular design approach, enabling the design of personalized drug-release profiles, and demonstrates superior mechanical strength and tailored release kinetics compared with traditional methods. The core-shell design, characterized by high drug-loading capacity and a double-layered shell, ensured sustained drug release while maintaining the structural integrity of the formulation. At the same time, the external drug-loading layer eliminated the release delay of the CS structure. Furthermore, the drying process, physicochemical properties, and microstructure related to drug release were investigated and the quality and performance of the formulation were confirmed. Optimization of the formulation and process parameters not only enhanced the mechanical strength of the formulation but also enabled the creation of individualized dissolution profiles. This study highlights the applicability of DIW within the pharmaceutical sector, presenting novel insights related to advancements in pharmaceutical production and the realm of personalized medicine. Meanwhile, a series of *in vivo* characteristics of 3D-printed DDSs scientific issues are yet to be addressed to achieve precise treatment. Furthermore, *in vitro* and *in vivo* correlations through structure and personalized manufacturing are warranted.

## CRediT authorship contribution statement

**Zhiting Liu:** Writing – original draft, Supervision, Methodology, Investigation, Data curation. **Jiaying Huang:** Validation, Software, Methodology, Formal analysis, Data curation. **Danqiao Fang:** Visualization, Resources, Investigation. **Bohua Feng:** Validation, Project administration, Funding acquisition. **Jianxu Luo:** Supervision, Software, Resources. **Peixuan Lei:** Visualization, Formal analysis. **Xiaoling Chen:** Resources, Investigation, Data curation. **Qingchun Xie:** Project administration, Funding acquisition, Conceptualization. **Meiwan Chen:** Writing – review & editing, Visualization, Validation. **Peihong Chen:** Writing – review & editing, Writing – original draft, Project administration, Methodology, Conceptualization.

## Declaration of competing interest

The authors declare that they have no known competing financial interests or personal relationships that could have appeared to influence the work reported in this paper.

## Data Availability

Data will be made available on request.
