# Supplementary material for: Material extrusion 3D-printing technology: A new strategy for constructing water-soluble, high-dose, sustained-release drug formulations
Source: Mater Today Bio. 2024 Jul 14;27:101153. doi: 10.1016/j.mtbio.2024.101153 (PMC11287018; doi:10.1016/j.mtbio.2024.101153)
Supplement: Multimedia component 1 [file mmc1.docx]

Supporting information

Supporting Table 1. Parameters for 3D printing and slicing.

|  | MH-CS | MH-SS |
| --- | --- | --- |
| Z-offset action | 0.45 mm | 0.30 mm |
| Strand hight | 0.41 mm | 0.26 mm |
| Printing speed | 15.00 mm/s | 15.00 mm/s |
| Strand width | 0.41 mm | 0.26 mm |
| Infill distance | 0.45 mm | 0.26 mm |
| Outline Offset | 0.00 mm | 0.00 mm |
| Start break | 0.05 s | 0.05 s |
| End break | 0.05 s | 0.10 s |
| Layer angle change | 90° | 90° |
| Temperature | 25℃ | 40℃ |
| Nozzle size | 0.41 mm | 0.26 mm |

Supporting Table 2. Dimensions of the digital prototype and final MH-CS model.

|  | 250 mg MH-CS | | 500 mg MH-CS | |
| --- | --- | --- | --- | --- |
|  | Digital (mm) | Actual (mm) | Digital (mm) | Actual (mm) |
| long axis (mm) | 13.00 | 13.12 ± 0.06 | 17.30 | 17.41 ± 0.03 |
| Short axis (mm) | 6.00 | 6.10 ± 0.04 | 7.80 | 8.05 ± 0.01 |
| Height(mm) | 4.00 | 3.92 ± 0.02 | 4.30 | 4.25 ± 0.03 |

Supporting Table 3. The weight of MH-CS.

|  | 250 mg | 500 mg |
| --- | --- | --- |
| Weight (mg) | 269.80 ± 3.20 | 521.10 ± 4.20 |
| RSD (%) | 1.19 | 0.81 |

Supporting Table 4. Drug content of 250-mg MH-CS.

| Bath. No | Drug content (mg) | Drug loading  (%) | Marked content  (%) |
| --- | --- | --- | --- |
| MH-CS 211021250 | 254.27 | 95.87 | 101.71 |
| MH-CS 211022250 | 254.79 | 95.26 | 101.92 |
| MH-CS 211023250 | 256.28 | 96.74 | 102.51 |
| Average | 255.11 ± 0.85  (RSD = 0.33%) | 95.96 ± 0.60  (RSD = 0.63%) | 102.05 ± 0.34  (RSD = 0.33%) |

Supporting Table 5. Drug content of 500-mg MH-CS.

| Bath. No | Drug content (mg) | Drug loading  (%) | | Marked content  (%) |
| --- | --- | --- | --- | --- |
| MH-CS 211021500 | 499.51 | | 95.56 | 99.90 |
| MH-CS 211022500 | 501.00 | | 95.90 | 100.20 |
| MH-CS 211023500 | 500.60 | | 95.21 | 100.12 |
| Average | 500.37 ± 0.63 (RSD = 0.13%) | | 95.56 ± 0.28  (RSD = 0.29%) | 100.07 ± 0.13  (RSD = 0.13%) |

Supporting Table 6. Influence of plasticizers on printing pressure in DIW processes.

| Formulation | F_PEG_ | F_TEC_ | F_GLY_ |
| --- | --- | --- | --- |
| Printing pressure (kPa) | 430 ± 5 | 440 ± 5 | 510 ± 5 |

Supporting Table 7. Final formulation composition of MH-CSS.

| MH-CS | | | MH-SS | |
| --- | --- | --- | --- | --- |
| Component | Content (w/w) | | Component | Content (w/w) |
| MH | 96.0% | | MH | 4.0% |
| PVP k90 | 4.0% | RL : RS (4∶1) | | 36.8% |
| Solvent | Ethanol | | Talc | 50.0% |
| / | / | | TEC | 9.2% |
| / | / | | Solvent | 95% Ethanol |

Supporting Table 8. The weight of MH-CSS.

|  | 250-mg MH-CSS | 500-mg MH-CSS |
| --- | --- | --- |
| Weight (mg) | 412.72 ± 2.76 | 787.60 ±7.9 |
| RSD (%) | 0.67 | 1.00 |

Supporting Table 9. Dimensions of the digital prototype and final MH-CSS model.

|  | 250-mg MH-CSS | | 500-mg MH-CSS | |
| --- | --- | --- | --- | --- |
|  | Digital (mm) | Actual (mm) | Digital (mm) | Actual (mm) |
| long axis (mm) | 15.00 | 15.12 ± 0.02 | 17.80 | 17.88 ± 0.03 |
| Short axis (mm) | 6.25 | 6.34 ± 0.03 | 8.60 | 8.71 ± 0.02 |
| Height (mm) | 4.75 | 4.69 ± 0.02 | 5.30 | 5.18 ± 0.02 |

Supporting Table 10. Drug content of 250-mg MH-CSS.

| Bath. No | Drug content  (mg) | Drug loading  (%) | Marked content  (%) |
| --- | --- | --- | --- |
| MH-CSS 221120250 | 250.38 | 61.20 | 100.15 |
| MH-CSS 221121250 | 256.51 | 61.37 | 102.60 |
| MH-CSS 221122250 | 258.45 | 61.84 | 103.38 |
| Average | 255.11 ± 3.44  (RSD = 1.35%) | 61.47 ± 0.26  (RSD = 0.42%) | 102.05 ± 1.29  (RSD = 1.27%) |

Supporting Table 11. Drug content of 500-mg MH-CSS.

| Bath. No | Drug content  (mg) | Drug loading  (%) | Marked content  (%) |
| --- | --- | --- | --- |
| MH-CSS 221120500 | 503.61 | 64.57 | 100.72 |
| MH-CSS 221121500 | 507.10 | 64.19 | 101.42 |
| MH-CSS 221122500 | 500.39 | 64.99 | 100.08 |
| Average | 503.70 ± 2.74  (RSD = 0.54%) | 64.58 ± 0.32  (RSD = 0.50%) | 100.74 ± 0.55  (RSD = 0.54%) |

Supporting Table 12. Fitting of release models for MH-CSS and RP.

| Formulation | Zero order  (R^2^) | | First order (R^2^) | Higuchi (R^2^) | Korsmeyer-Peppas (R^2^) | n value |
| --- | --- | --- | --- | --- | --- | --- |
| 250-mg MH-CSS | 0.9568 | 0.9864 | | 0.9910 | 0.9939 | 0.6188 |
| 500-mg MH-CSS | 0.9905 | 0.9701 | | 0.9727 | 0.9654 | 0.5802 |
| 500-mg MH-CSS (Model I) | 0.9809 | 0.8632 | | 0.9809 | 0.8712 | 0.3985 |
| RP | 0.8793 | 0.9923 | | 0.9742 | 0.9797 | 0.5207 |


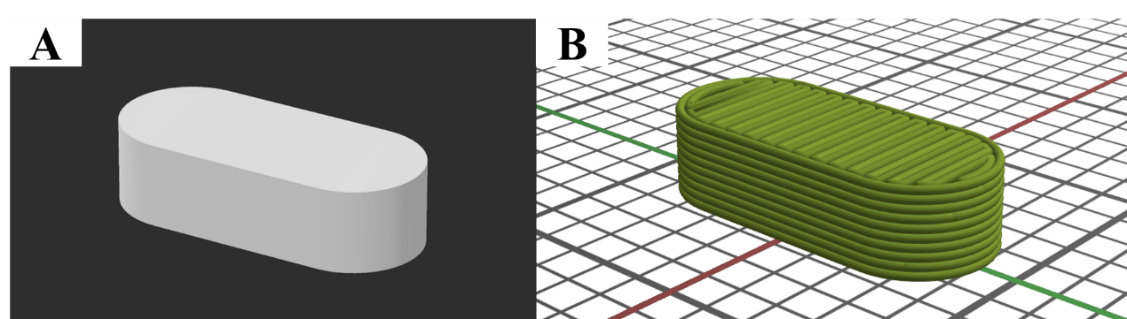


Supporting Figure 1. (A) Digital model of MH - CS and (B) Slicing model of MH – CS.


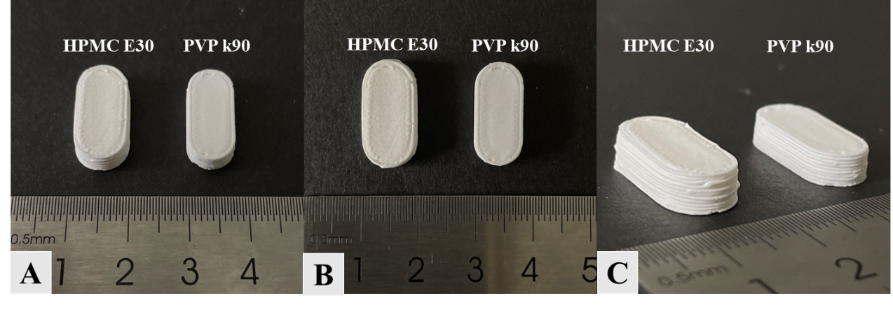


Supporting Figure 2. Appearance of F_6%E30_ and F_6%k90_ (A) Front view (B) Top view (C) Side view.


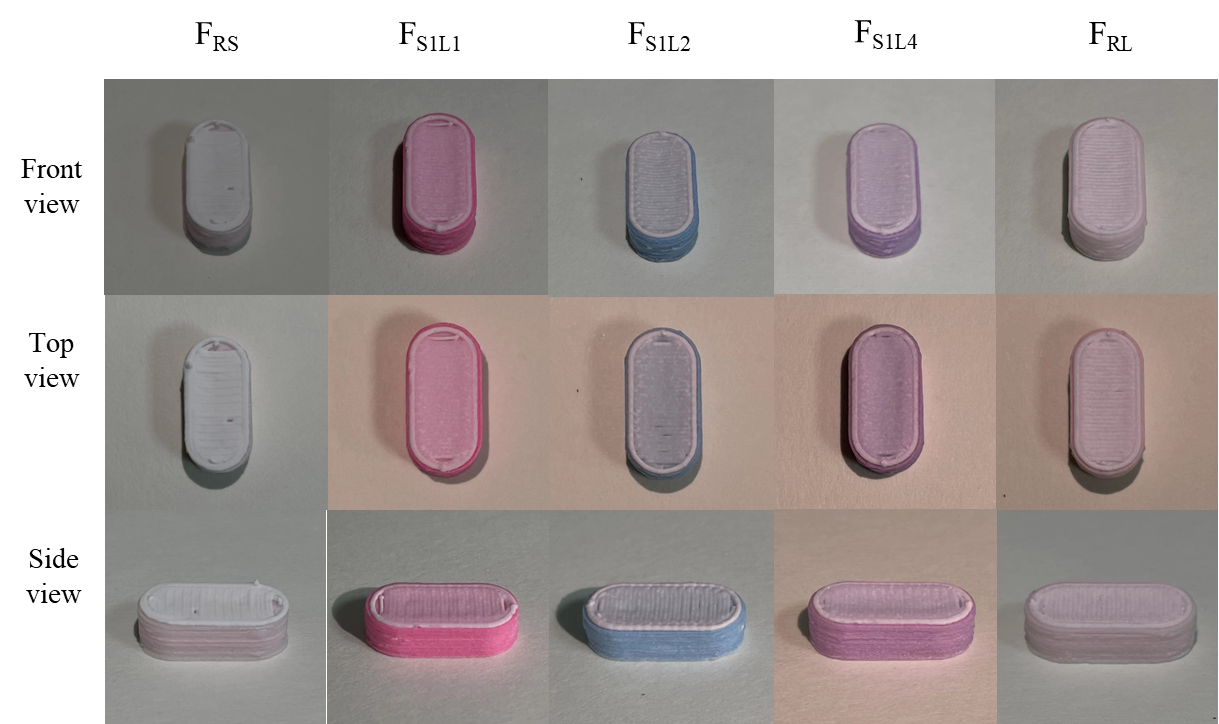


Supporting Figure 3. The effect of RL and RS ratio on the appearance of MH-CSS.


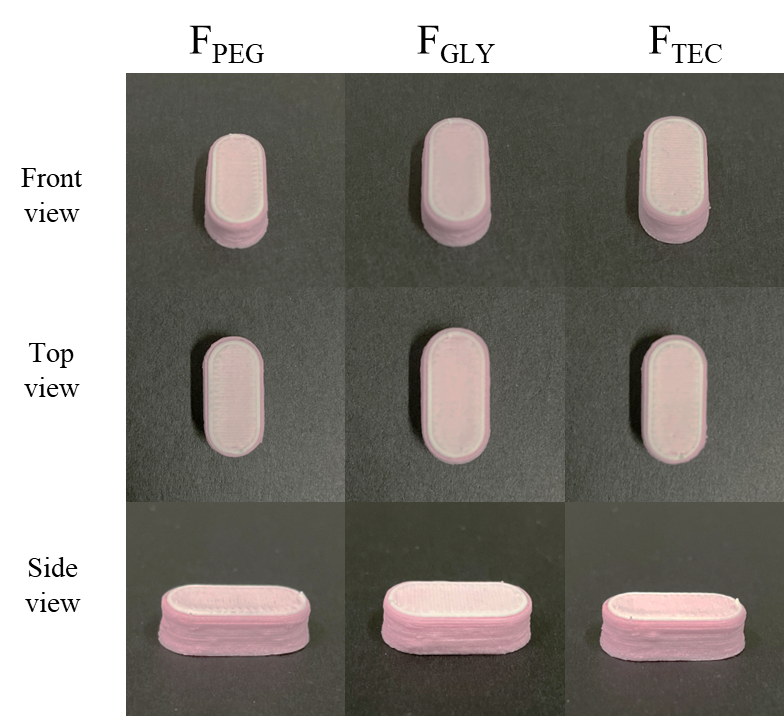


Supporting Figure 4. The effect of plasticizers on the appearance of MH-CSS.


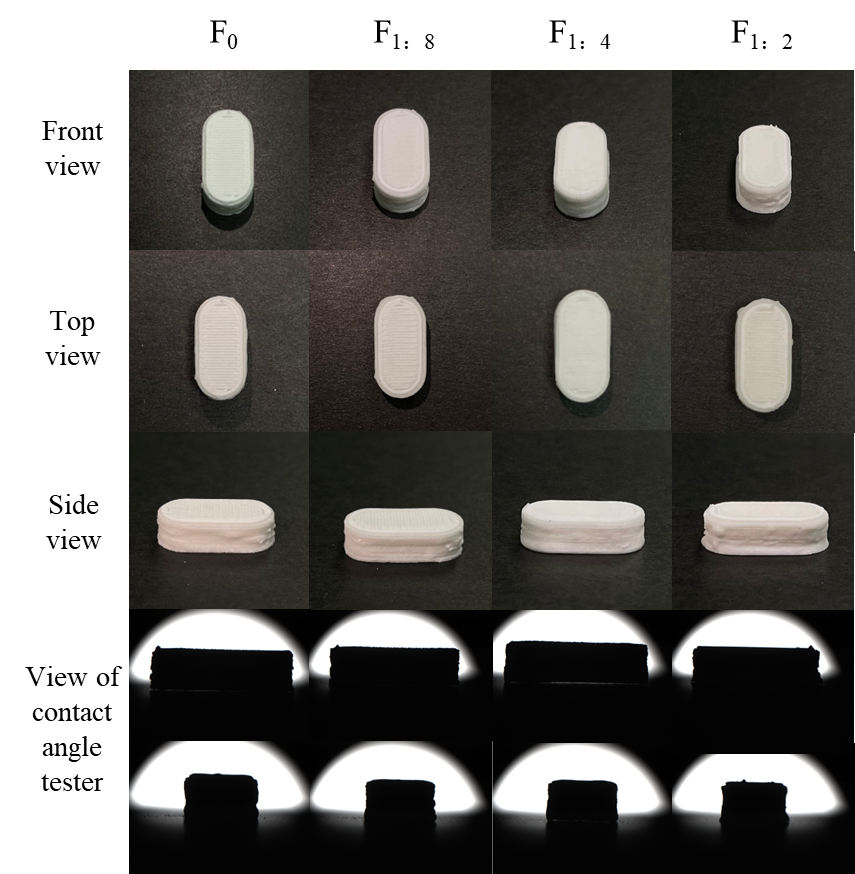


Supporting Figure 5. The effect of TEC dosage on MH-CSS appearance.


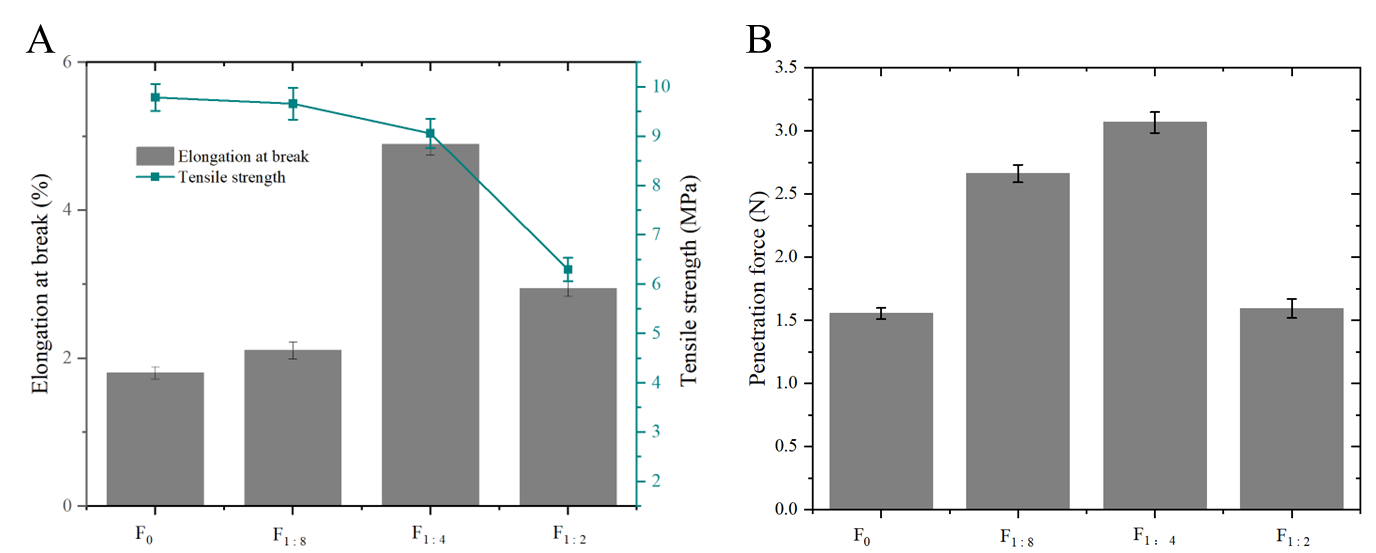


Supporting Figure 6. Mechanical properties of MH-SS (A) Tensile (B) Puncture force.


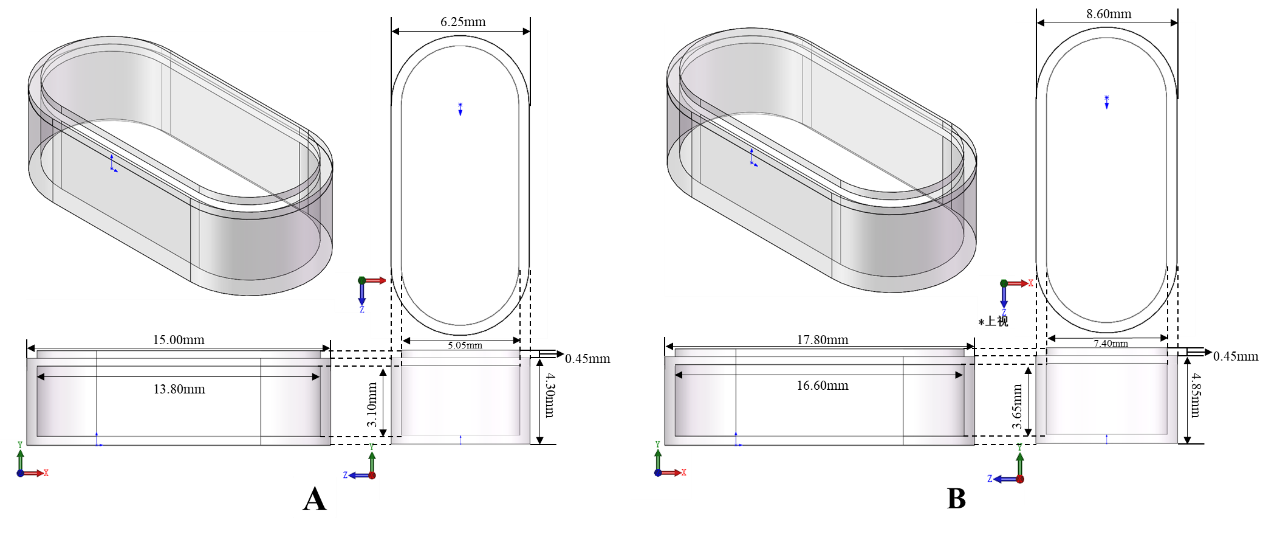


Supporting Figure 7. 3D models of (A) 250-mg and (B) 500-mg MH-CSS.
